# Crustal deformation, active tectonics and seismic potential in the Sicily Channel (Central Mediterranean), along the Nubia–Eurasia plate boundary

**DOI:** 10.1038/s41598-020-78063-1

**Published:** 2020-12-04

**Authors:** Mimmo Palano, Andrea Ursino, Salvatore Spampinato, Federica Sparacino, Alina Polonia, Luca Gasperini

**Affiliations:** 1 Istituto Nazionale di Geofisica e Vulcanologia, Sezione di Catania - Osservatorio Etneo, Piazza Roma 2, 95125 Catania, Italy; 2 Institute of Marine Sciences, CNR ISMAR-Bo, Via P. Gobetti 101, 40129 Bologna, Italy

**Keywords:** Solid Earth sciences, Geodynamics, Geophysics, Tectonics

## Abstract

Based on multidisciplinary data, including seismological and geodetic observations, as well as seismic reflection profiles and gravity maps, we analysed the pattern of crustal deformation and active tectonics in the Sicily Channel, a key observation point to unravel the complex interaction between two major plates, Nubia and Eurasia, in the Mediterranean Sea. Our data highlight the presence of an active ~ 220-km-long complex lithospheric fault system (here named the Lampedusa-Sciacca Shear Zone), approximately oriented N–S, crossing the study area with left-lateral strike-slip deformations, active volcanism and high heat flow. We suggest that this shear zone represents the most active tectonic domain in the area, while the NW–SE elongated rifting pattern, considered the first order tectonic feature, appears currently inactive and sealed by undeformed recent (Lower Pleistocene?) deposits. Estimates of seismological and geodetic moment-rates, 6.58 × 10^15^ Nm/year and 7.24 × 10^17^ Nm/year, respectively, suggests that seismicity accounts only for ~ 0.9% of crustal deformation, while the anomalous thermal state and the low thickness of the crust would significantly inhibit frictional sliding in favour of creeping and aseismic deformation. We therefore conclude that a significant amount of the estimated crustal deformation-rate occurs aseismically, opening new scenarios for seismic risk assessments in the region.

## Introduction

Comparisons between seismological and geodetic deformation-rates may provide significant insights for seismic hazard assessment in tectonically active regions. Moment-rates budgets based on geodetic observations capture both anelastic and elastic crustal deformations, while those estimated by seismological data are only sensitive to brittle slip along active faults. This comparison allows identifying regions where deformation is entirely released by crustal seismicity from those where the excess of deformation can be released either through large impending earthquakes or aseismic slip across creeping faults. Examples where this comparison has been successfully applied encompass several tectonic regions worldwide, including Iran^1,2^, western Canada^3^, western USA^4^, Greece^5^ and the western Mediterranean^6^.

**Figure 1:**
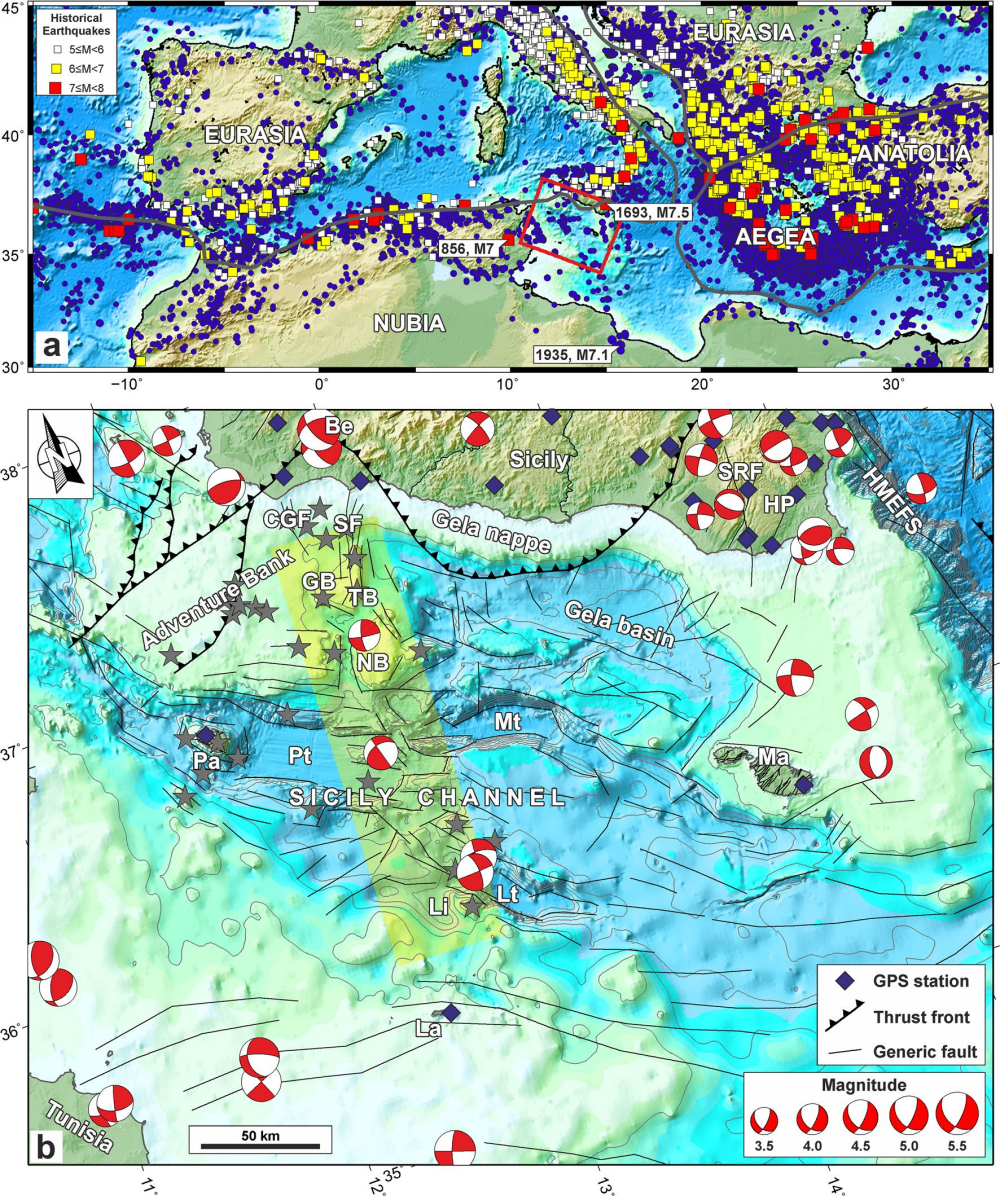
(**a**) Simplified map of the Mediterranean region. Main plate boundaries are marked by the grey line. The instrumental crustal seismicity (depth 0–50 km) with M ≥ 4 occurring during the 1905–2019 interval (http://www.isc.ac.uk/iscbulletin/search/catalogue/) is reported as blue points. The historical earthquakes with estimated magnitude M ≥ 5 during the 856–1904 interval^8,9^ (https://www.emidius.eu/SHEEC/) is reported as coloured square. The red box represents the region reported in panel (**b**). (**b**) Simplified tectonic map of the Sicily Channel and surrounding areas (the map is plotted in an oblique Mercator projection). Focal mechanisms of earthquakes with M > 3.5 are also reported^10^ (http://www.bo.ingv.it/RCMT/searchRCMT.html). The yellow strip represents the N-S-oriented tectonic belt discussed in the main text. Subaerial and submerged volcanic edifices^11–13^ are reported as red stars. *La* Lampedusa, *Li* Linosa, *Pa* Pantelleria, *Ma* Malta, *Lt* Linosa trough, *Mt* Malta trough, *Pt* Pantelleria trough, *NB* Nameless Bank, *TB* Terribile Bank, *GB* Graham Bank, *HP* Hyblean Plateau, *HMEFS* Hyblean Maltese Escarpment fault system, *CGF* Capo Granitola fault, *SF* Sciacca fault. Maps compiled using the Generic Mapping Tool, version 5^14^; image editing using Inkscape, version 1 (https://inkscape.org).

This approach could also be attempted in regions characterized by composite deformation patterns, such as the Sicily Channel in the central Mediterranean (Fig. 1a), where the Nubia–Eurasia convergence is accommodated by geometrically complex boundaries characterized by multiphase tectonics and relatively slow deformation rates (~ 7.5 mm/year as predicted by the 3.16-Myr-average MORVEL Nubia–Eurasia pole^7^).

The Sicily Channel is part of the Pelagian block^15–17^, a 25–30 km thick continental crustal portion of the Nubian continental margin, which extends from the Sahel region of Tunisia to eastern Sicily, and is separated from the Ionian basin by a regional tectonic boundary named the Hyblean-Maltese Escarpment fault system (Fig. 1b). The tectonic configuration of the Pelagian block includes a series of mostly WNW-ESE trending structural highs and basins, bordered by variously oriented faults of Neogene-Quaternary age.

Evolution of the Pelagian block has been strongly influenced by the complex Nubia–Eurasia plate interaction (see details in Supplementary Information), with considerable changes in structural styles, convergence attitude and deformation rates since the Late Cretaceous^18^. Starting from the Late Miocene and mostly during the Early Pliocene, a lithospheric-scale continental rifting occurred in the central part of the Pelagian block^19^, with a subsequent phase (Late Pliocene–Pleistocene) characterized by a magma-assisted extension^20^. This rifting process led to the development of NW–SE-trending tectonic depressions (e.g., Pantelleria, Linosa and Malta troughs), bordered by crustal normal faults with variable throws^21^ (Fig. 1b). A broad N-S-oriented belt, defined on the basis of tectonic bathymetric, volcanic and magnetic lineaments and extending from Lampedusa Island to the Graham Bank^11,22,23^ (Fig. 1b), separates the rift system in two sectors: the Pantelleria trough to the west, and the Malta and Linosa troughs to the east (Fig. 1b). Along this deformation belt, the magmatic activity started in the Nameless Bank in the Late Miocene and continued to the present-day close to Pantelleria and to the south-eastern wedge of the Graham Bank^12,24^ and is characterized by a wide spectrum of volcanic rocks with tholeiitic, alkaline and peralkaline affinities^25^ (see details in Supplementary Information).

Previous studies have also highlighted that instrumental seismicity mainly concentrates along the N-S-oriented belt and is characterized by moderate levels of seismic energy release (magnitude up to 4.7), with predominance of earthquakes at 10–20 km of depth, and occasionally deeper events^26,27^. Nevertheless, the occurrence of large earthquakes (M > 7) during historical times on nearby regions (e.g., the 856^8^ M7, the 1693^28^ M7.5 and the 1935^29^ M7.1 earthquakes striking the Tunisia, the Hyblean Plateau and the offshore of NW Libya, respectively) would suggest, for Sicily Channel sector, a greater seismic hazard than that currently expected^30^.

Available focal mechanisms^10^ (http://www.bo.ingv.it/RCMT/searchRCMT.html), although poorly distributed, highlight a prevailing strike-slip regime in the Sicily Channel region (Fig. 1b), while further information on active tectonics have been provided from geodetic data^31^, which show a composite pattern of extension (~ 1.4 mm/year) and contraction (~ 2.9 mm/year) along the NE–SW and NW–SE directions, respectively. However, the present-day tectonic setting is still a matter of debate, which could be better addressed through the analysis of geodetic and seismological data integrated by geo-structural information.

In this work, we performed a comparison between geodetic moment-rate, based on a combination of GPS (Global Positioning System) observations, and seismic moment-rate from earthquake catalogues, aiming to statistically evaluate the deformation-rate budget for the sector of the Sicily Channel which is currently characterized by significant tectonic activity (Fig. 1b). Results have been framed within the current tectonic setting derived by morpho-bathymetric, seismic reflection and gravity data collected in the last decades.

## Results

### Seismology

**Figure 2:**
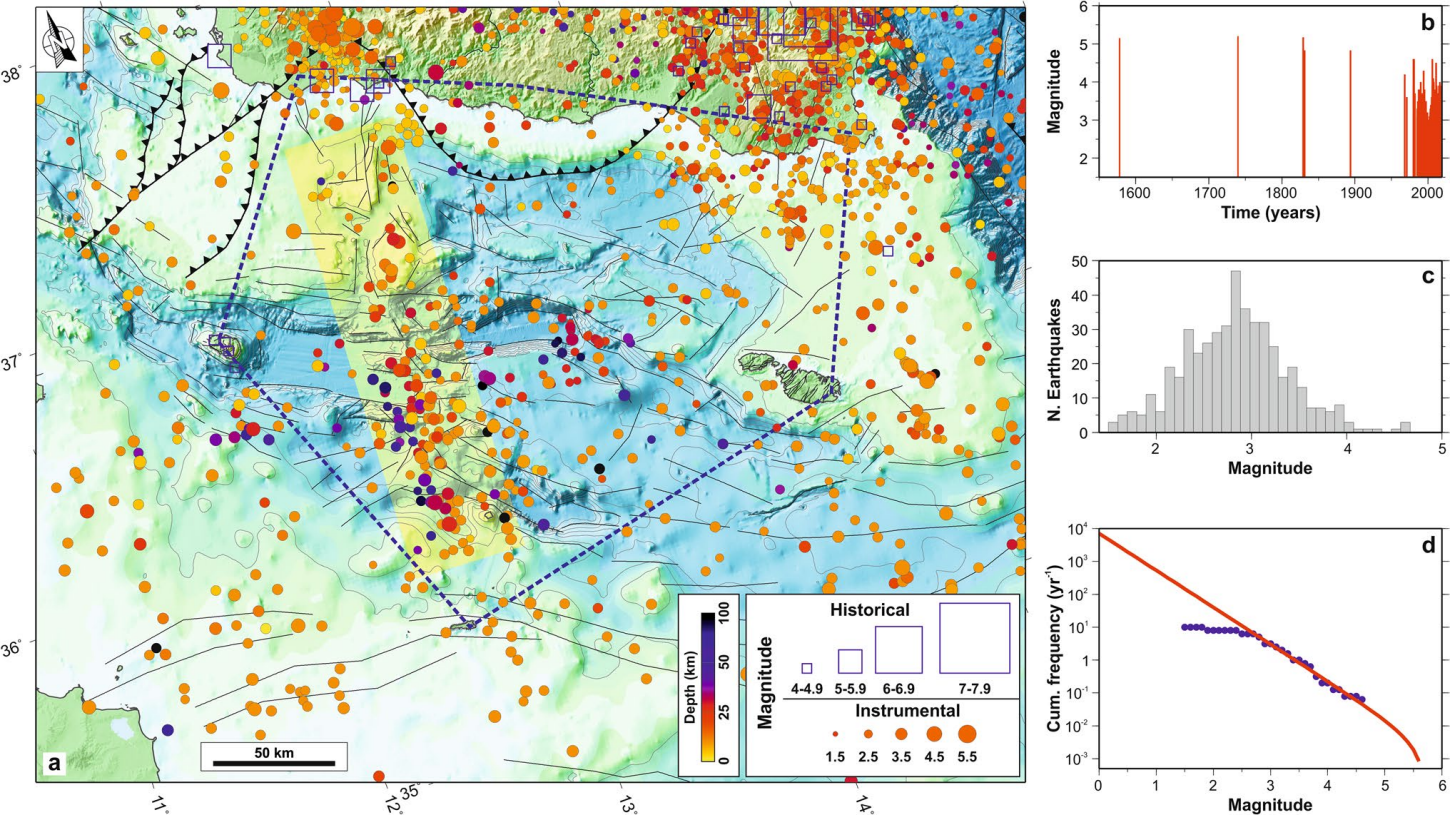
(**a**) Instrumental (circle) and historical (square) seismicity of northern Sicily Channel. The instrumental seismicity covers the 1966–2018 interval and has been collected from various sources (http://www.isc.ac.uk/iscbulletin/search/catalogue/; http://iside.rm.ingv.it). The yellow strip represents the N-S tectonic belt. The blue polygon defines the study area; inside this area, the historical seismicity covers the 1578–1965 time interval (https://www.emidius.eu/SHEEC/). (**b**) Temporal trend of historical and instrumental seismicity for the study area (blue polygon in panel a). (**c**) Magnitude range histogram for instrumental seismicity striking the study area. (**d**) Cumulative frequency-magnitude distributions (blue points) of earthquakes for the study area. The red line represents the truncated Gutenberg-Richter formulation^34^ (see also Table 1). Maps compiled using the Generic Mapping Tool, version 5^14^; image editing using Inkscape, version 1 (https://inkscape.org).

Data from instrumental seismicity catalogues (see “Data and Methods” section) highlight main seismic features in the Sicily Channel region, which, despite the presence of active faults and its composite geodetic deformation, is characterized by a low-to-moderate seismic activity^27,32^ in comparison with the one observed (Fig. 1a) on nearby regions such as Sicily^28^ and North Africa^8,9,29^, located along the Nubia–Eurasia convergent plate boundary. In detail, in the Sicily Channel, instrumental seismicity is scant and mainly concentrated along the above mentioned N-S-oriented belt^26,27,32,33^ (Fig. 2a). Conversely, in southern Sicily, seismicity is mainly clustered in the Hyblean plateau and the Belice area, and marks the presence of active tectonic structures which were the site of historical earthquakes (Fig. 2a). Historical catalogues document that, for the whole region (Fig. 2a), large seismic events (M ≥ 6.5) have taken place since 1125 (https://www.emidius.eu/SHEEC/).

**Table 1:** Summary of adopted (*M*_*x*_*, H*_*s*_*, A, μ*) and estimated (*M*_*c*_, *a*, *b*, $\dot{M}_{seis}$, $\dot{M}_{geod}$) parameter values.

*M*_*x*_	5.7
*H*_*s*_	13 km
*A*	4.1 × 10^10^ m^2^
*μ*	3 × 10^10^ N/m^2^
*M*_*c*_	2.8 ± 0.2
*a*	3.85 ± 0.11
*b*	1.12 ± 0.08
$\dot{M}_{seis}$	6.58 × 10^15^ Nm/year
$\dot{M}_{geod}$	7.24 × 10^17^ Nm/year

Our statistical evaluation of the deformation-rate budget for the Sicily Channel was focused on the area delimited by the blue polygon in Fig. 2a, which was chosen in relation to the distribution of continuous GPS stations. In this area, the SHEEC catalogue reports the occurrence of moderate earthquakes (M > 4.5) only since 1578 (Fig. 2b), close to Pantelleria and along the south-western Sicily coast (Fig. 2a). The seismic moment-rate was calculated according to Eq. 1 (see “Data and Methods” section), which implies a seismic moment-rate estimate dependent on the largest magnitude value (*M*_*x*_) in the area. The simplest method of calculating *M*_*x*_ is performed considering the largest earthquake reported in the seismic catalogue and by adding 0.5^35^. The largest earthquake striking our study area (Fig. 2a) took place in 1740, with an estimated magnitude of 5.2 (https://www.emidius.eu/SHEEC/). Therefore, we assumed a value of 5.7 as a maximum potential magnitude. Definition of *M*_*x*_ is a critical aspect for a robust seismic moment-rate estimate. By using the MMAX toolbox^36^, we performed some statistical estimates of *M*_*x*_ under different circumstances (completeness and temporal length of the catalogue, magnitude distribution and uncertainties, number of earthquakes, etc.), by adopting a wide spectrum of statistical procedures. The statistical estimations of *M*_*x*_ have been made by considering all historical and instrumental earthquakes with M ≥ 4.0. Achieved magnitude values range in the interval 5.22 (± 0.47) − 5.52 (± 0.44). The assumed value of potential magnitude 5.7 is at the upper boundary of these estimates, and therefore was considered a suitable estimate for the investigated region.

Under these assumptions and considering Eq. 1, our seismic moment-rate estimate for the study area is 6.58 × 10^15^ Nm/year (Table 1).

### Geodetic data

**Figure 3:**
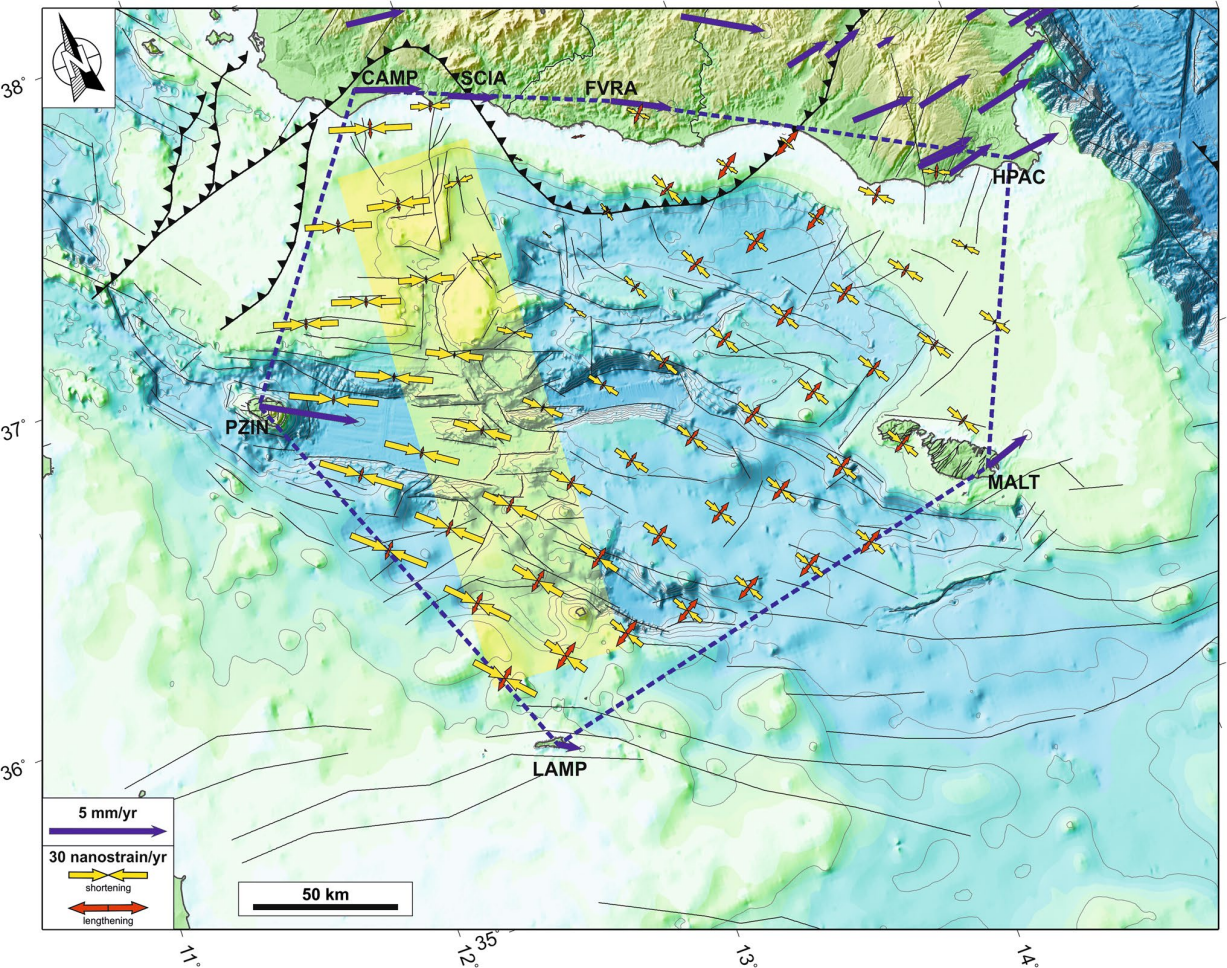
Estimated GPS velocities and associated uncertainties (at the 95% level of confidence) are reported as blue arrows. Velocities refer to a Nubian-fixed reference frame^37^. The geodetic horizontal strain-rate field (red and yellow arrows indicate the greatest extensional and contractional strain-rates, respectively) as estimated for the area defined by the blue polygon is also reported. Maps compiled using the Generic Mapping Tool, version 5^14^; image editing using Inkscape, version 1 (https://inkscape.org).

GPS observations acquired in the 2001.0–2018.0 time-interval from continuous stations located around the Sicily Channel and southern Sicily have been analysed to describe the current crustal deformation in the study area. Estimated GPS velocities, referred to a Nubia-fixed reference frame^37^, and associated uncertainties (at 95% level of confidence) are reported in Fig. 3. Within this frame, the station LAMP (Lampedusa, Fig. 3) shows a residual velocity of ~ 1 mm/year towards SSE, evidencing a small deviation from Nubia. Stations on the Hyblean-Malta block are moving toward ENE with rates of ~ 2.3 mm/year, while stations in Pantelleria (PZIN) and along the SW Sicilian onshore move eastward, with rates ranging between ~ 3.8 and 2.1 mm/year, respectively. The strain-rate field also suggests that the western sector of our study area (Fig. 2a) is dominated by a prevailing contractional field, with *ε*_*hmin*_ axes having a WNW-ESE orientation between Pantelleria and SW Sicily, and a NW–SE attitude between Pantelleria and Lampedusa. Conversely, the eastern sector is characterized by a strike-slip deformation field, with *ε*_*Hmax*_ and *ε*_*hmin*_ axes aligned to the NE-SW and to the NW–SE direction, respectively (Fig. 3). Assuming a value of 13 km^30^ as average seismogenic thickness *H*_*s*_, and according to Eq. 4 (see “Data and Methods” section), we estimated a geodetic moment-rate of 7.24 × 10^17^ Nm/year for the investigated area (Table 1).

### Seismic reflection profiles

**Figure 4:**
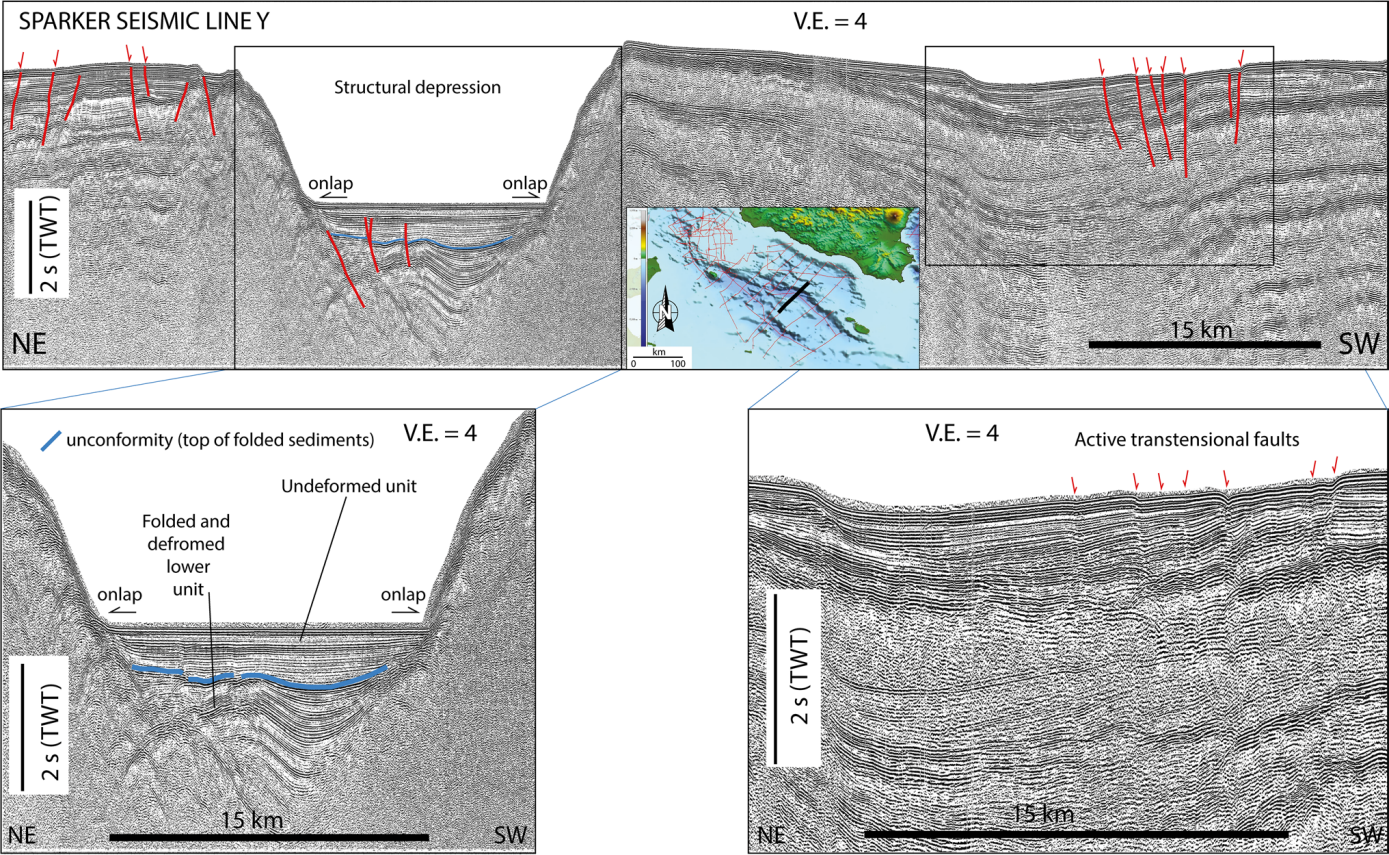
Seismic profile Y crossing orthogonally the deep through of the Sicily Channel. We note that reflector H1 (thick blue line) marks a major unconformity separating two stratigraphic units in the depocenter: the lower unit is intensely deformed, while the upper (U1) undisturbed unit onlaps the acoustic basement. Pervasive deformation of U1, in the form of subvertical trantensive faulting zones are only visible outside the basin depocentres, affecting the shoulders of the main rift. The images have been edited by using Adobe Illustrator CS6.

**Figure 5:**
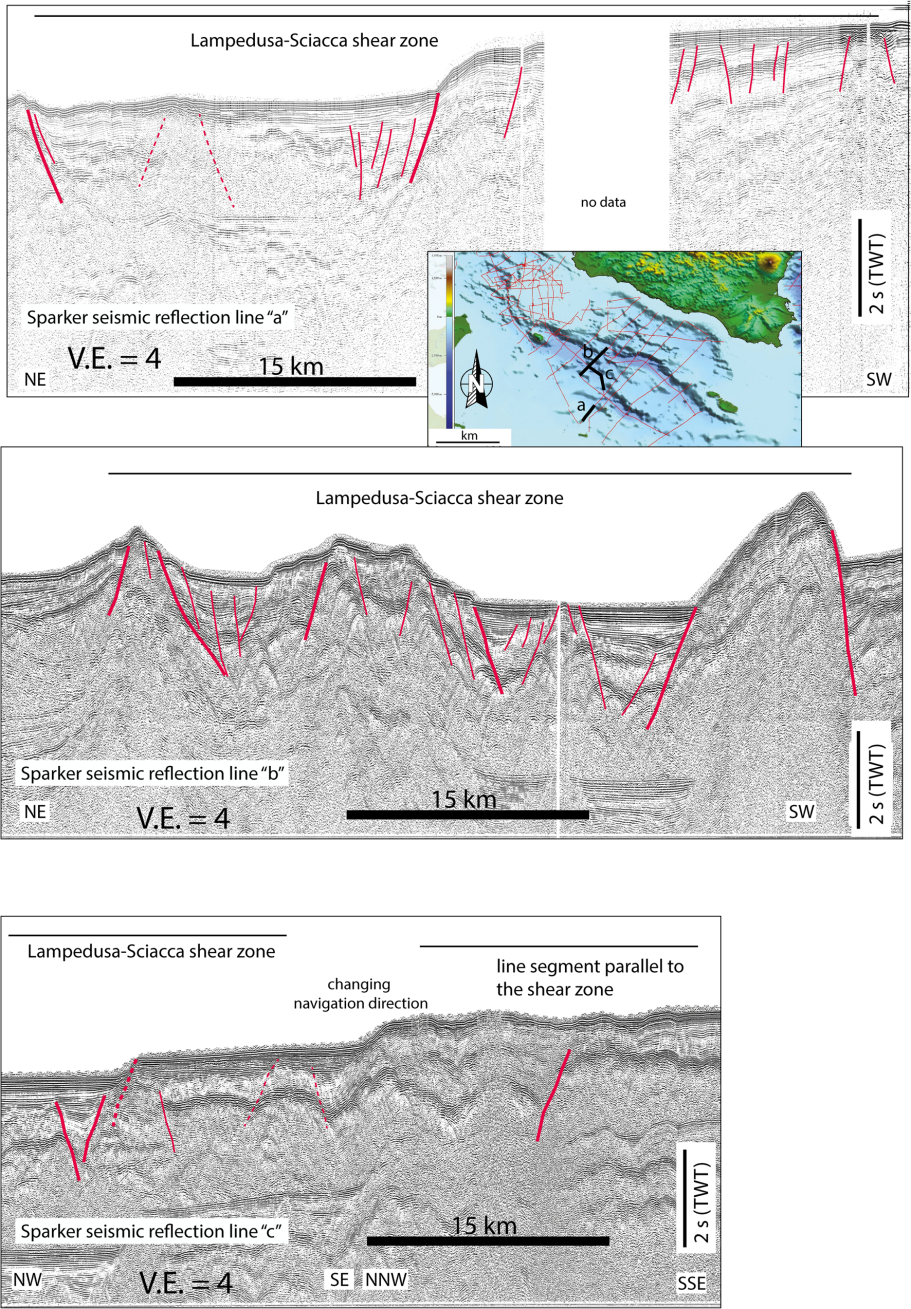
Seismic reflection profiles along the N-S corridor where strike-slip deformation has been detected. We note that faults are active reaching up to the seafloor, subvertical, and change their nature (transpression/transtension) laterally, as typically observed in wrench-tectonic domains. The images have been edited by using Adobe Illustrator CS6.

Based on the interpretation of marine geophysical data combined with the well-log stratigraphy of the ViDEPI project (https://www.videpi.com/videpi/videpi.asp), numerous papers have described tectonic structures accommodating multiphase crustal deformations in the Sicily Channel^23,38–45^. All these data and their interpretation have enabled describing a complex pattern of tectonic structures, which include active and inactivated features, as well as major changes in the geodynamic pattern. However, the lack of clear kinematic constraints, as well as evidence of the current activity, do not allow defining an unequivocal present-day seismo-tectonic setting. Here, taking advantage from geodetic and seismological results, we performed a new analysis of SPARKER seismic data acquired in the Sicily Channel during the 70’s (Figs. 4 and 5; see also Figs. S2 and S3 in the Supplementary Material), with the main purpose of distinguishing between active and inactive tectonic deformations. Seismic reflection profiles and line-drawing interpretations highlight the presence of a complex deformation pattern in the study area. We observed that the basin depocenters mark first order structural boundaries between different morpho-structural domains. However, seismic images suggest that the basin depocenters are not tectonically active, since the uppermost sedimentary deposits are not affected by incipient deformations, and onlap horizontally the basin margins (Fig. 4). Conversely, SPARKER profiles show evidences of incipient activity along the N–S shear zone, depicting a diffuse and complex pattern of transtensional and transpressional deformations, affecting the sedimentary sequence up to the seafloor (Fig. 5). Sediments in depocenters can be subdivided into two seismo-stratigraphic units, separated by a major unconformity (H1 in Fig. 4). A recent sediment layer of the upper unit appears relatively undeformed and shows only local evidence of sub-vertical faulting, never affecting the seafloor (Fig. 4). Based on available seismo-stratigraphic constraints, H1 might be correlated to a major Early Pliocene tectonic event, when fault-dominated extension shifted to a magma assisted rifting without a strong tectonic component^20^. On the other hand, chrono-stratigraphic well logs in the Gela basin (i.e., the Palma well whose data are reported in the ViDEPI project), suggest that reflector H1 observed in our seismic reflection profiles might correspond to a stratigraphic hiatus dated to the lower Pleistocene. However, we note that in the depocenters, H1 is located about 1 s two-way-time below the seafloor, roughly corresponding to about 1 km of depth. Considering a constant sedimentation rate of 1 mm/year, as deduced for the uppermost sedimentary sequence by radiometric dating^46^, this level might be dated back to about 1 Ma. This estimate, although very rough, agrees with chrono-stratigraphic and biostratigraphic reconstructions carried out in the Gela basin, where the more recent depositional sequence boundaries were dated to 1.4 Ma (Early Pleistocene) and 0.8 Ma, during a peak of the regression^43,47^. More detailed age constraints, which would allow to distinguish between different scenarios are not available to date, but we suggest that H1 could represent the end of the fault-guided rifting processes responsible for the development of the main tectonic depressions. Therefore, H1 should mark an abrupt change in the stress regime of the Sicily Channel region. Indeed, such an estimated age corresponds to a change in the Mediterranean geodynamics occurring as a consequence of a reduction of ca. 55% of the Nubia–Eurasia convergence rate^18^.

### Gravity maps

**Figure 6:**
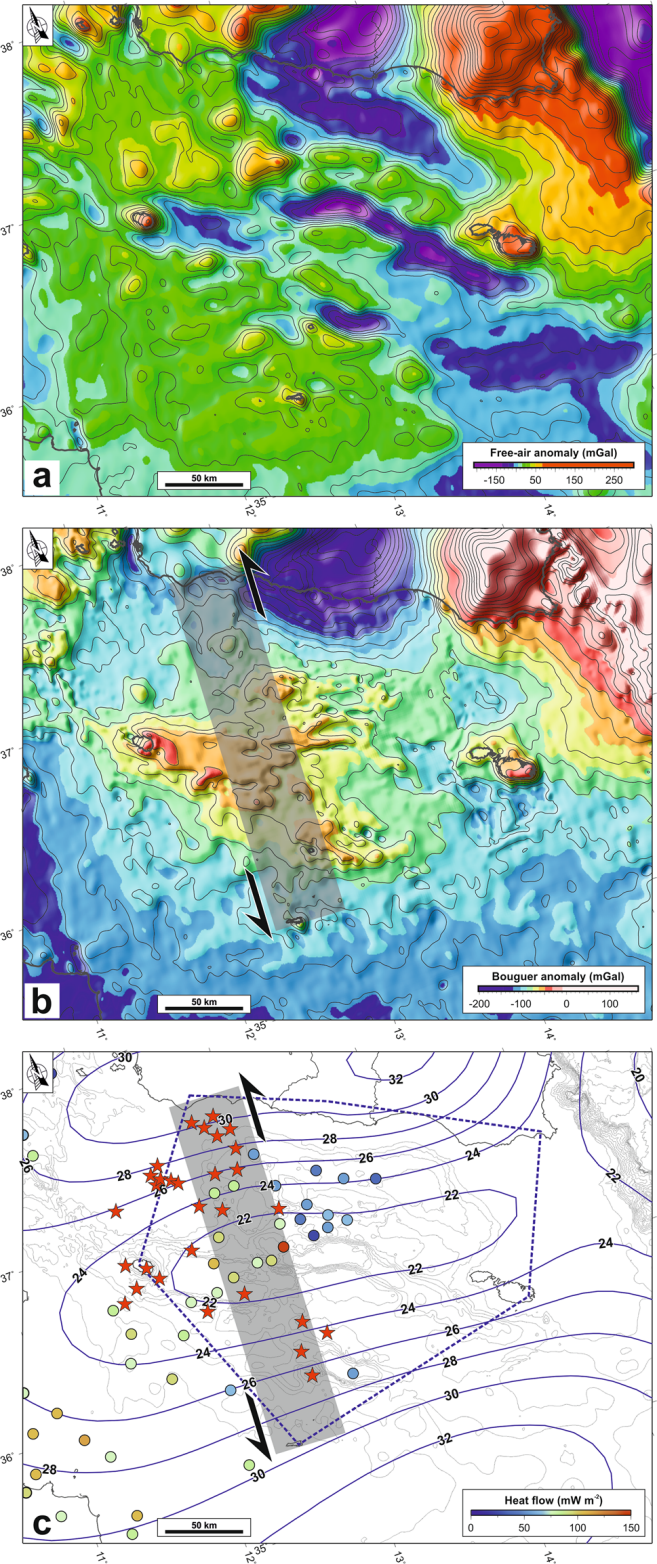
(**a**) Free-air gravity map. (**b**) Bouguer anomaly map. (**c**) Spatial distribution of surface heat flow measurements (coloured circles; from http://www.datapages.com/gis-map-publishing-program/gis-open-files/global-framework/global-heat-flow-database) and subaerial and submerged volcanic edifices^11–13^ (red stars). The crustal thickness^51^ (in km) is also reported as continuous blue lines. The gray strip represents the LSSZ. Maps compiled using the Generic Mapping Tool, version 5^14^; image editing using Inkscape, version 1 (https://inkscape.org).

Seismic reflection data available for the study area give insights into the shallow structural development, but suffer significant limitations. In fact, they are not homogeneously distributed and, in general, not oriented perpendicularly to the features under observation. For these reasons, mapping tectonic structures, especially those with an important strike-slip component, is challenging. Moreover, penetration of the seismic signal is relatively shallow, in general less than 1 km in the sedimentary sequence, thus seismic profiles are not able to image deformations affecting the acoustic basement. To overcome these problems, and in the attempt to gather structural information at the scale of seismological data, we carried out integrated analyses of gravity maps (Fig. 6a,b) compiled using the 29.1 release of satellite-derived data^48^ publicly available at https://topex.ucsd.edu/WWW_html/mar_grav.html. To compute the Bouguer correction, we adopted a Fast Fourier Transform approach^49^, employing bathymetric data from the EmodNet repository (https://www.emodnet.eu). The “gravfft” module of the Generic Mapping Tool software package^50^ was used for this purpose, considering densities of 1035 kg/m^3^ and 2700 kg/m^3^ for water and crust, respectively. The free-air gravity map of Fig. 6a highlights the presence of negative anomalies centred on the deep tectonic depressions which show NNW-SSE oriented axes. The map also highlights the presence of a major N-S transverse boundary displacing left-laterally major crustal features, which appears more evident in the Bouguer anomaly map (Fig. 6b).

### Robustness of geodetic and seismic moment-rate estimations

Geodetic and seismic moment-rate estimates are affected by some physical uncertainties. For instance, geodetic measurements should sample a time-interval long enough to: (i) minimize the effect of velocities uncertainties; and (ii) adequately sample both seismic and aseismic spectrum, as well as long-term deformation transients. Moreover, factors such as stations density, network geometry and smoothing parameters chosen for strain-rate estimates also affect the resulting geodetic moment-rates. Seismic moment-rate estimates are commonly affected by the completeness (i.e. all the earthquakes above a given magnitude should be fully reported) and the temporal length of seismic catalogues. Indeed, a relatively short time-interval (100–300 years) may not be representative of typical seismic cycles in a given region. To be considered robust, seismic moment-rate estimates performed using data from seismic catalogues require shorter average earthquake recurrence intervals than the catalogue duration^4^. On the other hand, instrumental 50–100 year-long catalogues are the most common source of data used worldwide in probabilistic seismic hazard analysis, under the assumption that such a time span would be adequate to derive earthquake return periods over timescales of 500–5000 years^3^.

**Figure 7:**
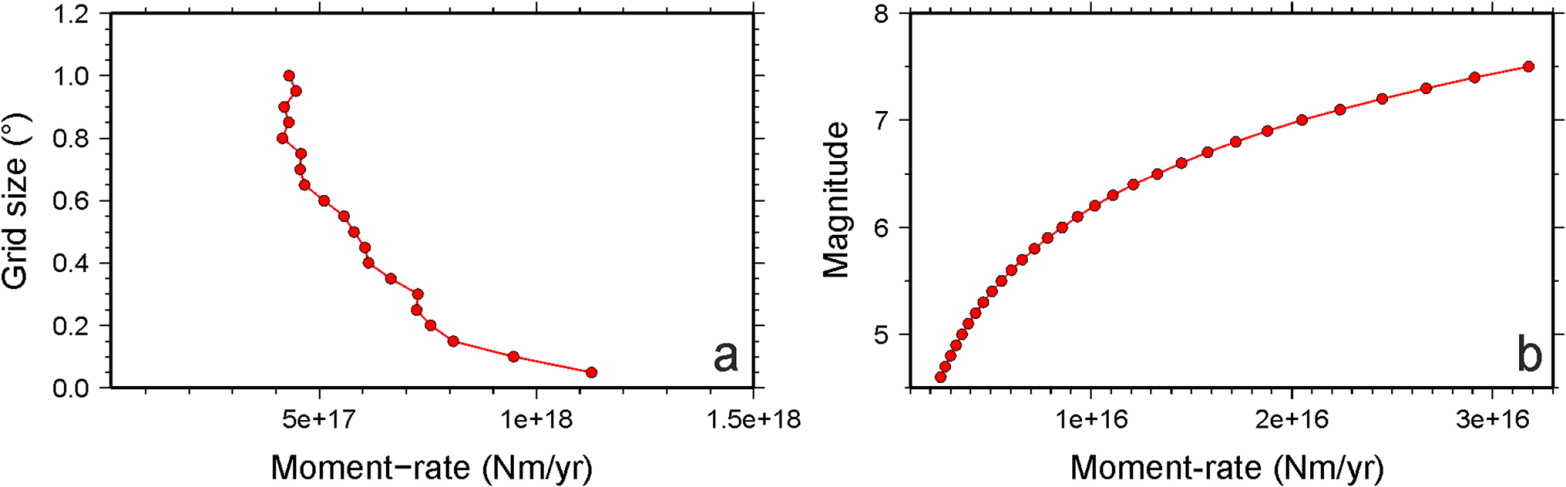
(**a**) Variation of geodetic moment-rate with respect to the size of the computational grid. (**b**) Variation of seismic moment-rate with respect to increasing *M*_*x*_ values. Maps compiled using the Generic Mapping Tool, version 5^14^.

Considering the above-mentioned factors, we performed some tests to assess the robustness of our estimates. First, we calculated additional strain-rate fields by simply varying the size of the computational grid (from 0.05° to 1.0°; see Supplementary Information). Results highlight that, as the grid size increases, the smoothing pattern and the number of local artefacts decrease (Fig. S1). Moreover, moment-rates estimates in the interval 1.13 × 10^18^–4.31 × 10^17^ Nm/year decrease as the computational grid size increases (Fig. 7a), its estimation being related to the largest value of strain-rate in the investigated region (Eq. 4). However, even considering the smallest value, the difference between geodetic and seismic moment-rates remains too large, as seismicity accounts only for 1.4% of the geodetic deformation. We performed additional estimations varying the seismogenic thickness *H*_*s*_ in the 9–13 km interval^30^. Results of this last test (Fig. S4 in Supplementary material) highlight that the geodetic moment-rate decreases according to the decrease of *H*_*s*_. Estimated values range in the interval 2.98 × 10^17^–1.13 × 10^18^ Nm/year. Considering again the smallest value, seismic deformation accounts only for ~ 2% of the geodetic one.

Regarding the seismic moment-rate, our seismic catalogue is temporally short with respect to the estimated return period for a wide area encompassing the investigated one^30,52^, so it might not be complete. To test this eventuality, as *a* and *b* parameters (Eq. 3) are well constrained, we did some additional tests by simply varying *M*_*x*_ in the 4.6–7.5 interval, where the lower value is the maximum magnitude reported in our instrumental catalogue, and the greater one represents the largest magnitude reported in the historical catalogue for the surrounding regions (the 1693 M7.5 earthquake striking the Hyblean Plateau; https://www.emidius.eu/SHEEC/). Results of this test (Fig. 7b) highlight that the seismic moment-rate increases according to the increase of *M*_*x*_. Estimated values range in the interval 2.51 × 10^15^–3.18 × 10^16^ Nm/year. Even considering the largest value, again the difference between the seismic moment-rate and the geodetic one remains large (seismicity accounts only for 4.4% of the geodetic deformation).

The performed tests clearly indicate that the observation of a significant mismatch between geodetic and seismic moment-rate is reliable.

## Discussion

Combining GPS observations and earthquake catalogues, we performed a statistical evaluation of the deformation-rate budget for the Sicily Channel area, which suggests that crustal seismicity accounts only for ~ 0.9% of the cumulated deformation. This implies a seismic moment deficit possibly covered by a portion of aseismic deformation (ongoing unloading by creep and other plastic processes) or by ongoing strain not yet released by seismicity (elastic storage).

Seismic reflection profiles analyses carried out in this study and those reported in the literature, have enabled us to provide an updated picture of the current geo-structural setting of the Sicily Channel. We note that, the NW-SE aligned tectonic depocentres are presently inactive, while seismic reflection profiles show evidences of active deformation along the N–S shear zone as highlighted by gravity, seismological and geodetic observations. Along this corridor, tectonic activity is marked by seafloor scarps, displaced seismic reflectors and chaotic sediment facies (Fig. 5). Moreover, it corresponds to an area where ongoing magmatic activity (e.g., subaerial and submerged volcanic edifices) has been described by several authors^11–13^, and where aligned patterns of magnetic anomalies suggest the widespread presence of magmatic bodies at different depths^11,53^.

The integrated analysis of available morpho-bathymetric data, seismic reflection profiles and gravity maps thus allows identifying a first-order structural feature, whose location, geometry and inferred kinematics, is in good agreement with the spatial distribution of recent volcanism and seismicity, therefore supporting the presence of a sub-vertical lithospheric shear zone favouring magma ascent^26^. Although the nature of this shear zone is poorly established, its deep roots and orientations inferred by gravity data, suggest that it might represent part of inherited Mesozoic discontinuities that cut the basement and formed along the rifted passive margin of the Tethys ocean^54,55^. These structural discontinuities acted as normal or transtensional faults until the Late Miocene, when they underwent transpressional reactivation^39,56–59^, but their recent activity seems accommodating the re-organization of the Nubia–Eurasia convergence along this segment of the plate boundary.

The observed N-S shear zone connects northward with two already described fault systems: (i) the Capo Granitola fault to the west; and (ii) the Sciacca fault system to the east. The former is made up of a sub-vertical master fault with few related splays, and extends for ca. 50 km with a N–S orientation from the offshore area of Capo Granitola to the volcanic area of the Graham Bank (Fig. 1b). The Capo Granitola fault, does not show clear evidence of present-day tectonic activity^20,42,45^, while the Sciacca fault, forming a positive flower structure, shows deformations reaching the sea-floor^42^.

Seismic reflection profiles analysed in this study do not show clear evidence of compressive deformation along the southern segment of the shear zone (Fig. 5). Older compressional features, such as folds and structural sediment undulations, call for a recent transtensional reactivation, as also suggested by the downthrow of seismic reflectors along high-angle structures (Fig. 5). This implies that the wide N-S shear zone is characterized by a complex pattern, including variable fault kinematics depending on relative orientation between pre-existing discontinuities and the present-day stress field. Compressional strain seems prevailing in the northern part of the shear zone^45,59^, while transtension characterizes a wide region to the south of the rifting depressions. This reconstruction however needs to be targeted by further analysis of geophysical data, which should combine high-resolution and deep penetration seismic images to investigate the deep tectonic control on the shallow structural development. Observed deformations define a ~ 220-km-long complex highly segmented lithospheric fault system that extends from Lampedusa to the SW Sicily offshore and shows prevailing left-lateral kinematics and named *Lampedusa—Sciacca shear zone* (LSSZ). Northward, the inland belt of Sicily is separated by a NNW-striking diffuse deformation zone separating the western and eastern belts. Along this diffuse deformation zone, oblique thrusting associated with clockwise rotations and wrench motions led to a differential shortening during the Neogene accretion of the Sicilian-Maghrebian thrust belt, therefore resulting in major rates in the eastern belts compared to the westernmost ones^55,60^. Along this diffuse deformation zone, an advective transfer through buried deep extensional faults linked to the mantle has been inferred on the basis of the occurrence of rising gas and hot waters enriched in mantle elements^61^ similarly to what was detected along major transcurrent/transform domains^62,63^. Moreover, recent tomographic studies imaged the presence of a deep discontinuity extending at least down to 30 km depth^26^, related also to a strong variation of the Moho depth, from 34 to 36 km below the eastern sector to less than 30 km below the western sector^55^. The same tectonic setting, with lithospheric connection between the lower plate mantle and upper plate structures, was described in the adjacent Ionian Sea, where a series of transverse/transtensional faults deeply fragmenting the convergent plate boundary trigger lower plate serpentinite diapirism^64^. Furthermore, this diffuse deformation zone together with the LSSZ might represent the current shallow expression of an inherited Mesozoic lithospheric discontinuity, formed along the rifted passive margin of the Tethys ocean. Such a discontinuity was involved in the last few Ma (Upper Neogene), into the Nubia–Eurasia convergence process, as suggested by the narrow indentation of the external front of the Sicilian-Maghrebian thrust belt (Fig. 1b).

Evidence of recent tectonic activity has been identified on the top of the Madrepore Bank and Malta High (Fig. 1b), in Late Quaternary deposits^43^. All these results clearly highlight the presence of several faults showing significant traces of activity during the Holocene, mainly along the LSSZ. Based on their deduced surface length (from 10 up to 50 km), these faults would be capable to generate earthquakes with magnitude values up to 7.2^65^. The use of scaling relations between the length of the fault and the maximum earthquake is widely used on regions where there are no historical data, but a number of issues arise when the fault planes are not exposed at the surface (i.e. buried and/or offshore faults) so that their geometry is constrained from regional seismic reflection profiles or from earthquake sequences and perhaps their length is poorly constrained. Beside these main problems, our deduced magnitude values agree well with the ones estimated for the Sicily Channel area by adopting other scaling relations^66^.

If crustal seismicity accounts for only ~ 0.9% of the cumulated deformation, in a region affected by active faulting capable of generating earthquakes with large magnitudes (M > 7), we need to re-evaluate the conditions for a reliable seismic hazard assessment to address the following questions: (i) is the lack of large earthquakes related to a longer return period than the observation time-span? (ii) will the excess of deformation be released through major impending earthquakes?

A return period of ca. 1000 years for M = 7.5 earthquakes has been estimated for a wide area including both the Hyblean Plateau and the Sicily Channel^52^. The moment-rate difference (Table 1) can be expressed in terms of the “missing” earthquake necessary to match the geodetic moment-rate. We therefore estimate that in the investigated crustal volume, an M = 5.8 earthquake is necessary each year to match the moment-rate difference. Alternatively, such a moment-rate difference can be filled by an M = 7.0 earthquake every 50 years or by an M = 7.5 earthquake every 310 years. This last estimate is ~ 3.2 times smaller than the return period reported in literature^52^. Although both instrumental and historical seismic catalogues correspond to a random sampling of the long-term seismicity pattern over the seismic cycle, it would be unrealistic to associate the moment-rate discrepancy only to a “missing” part of the earthquake catalogue. Indeed, available historical and instrumental catalogues suggest a scenario where a small portion of this moment-rate difference could be compensated by minor to moderate earthquakes. This agrees with the structural setting of the LSSZ as highlighted by seismic reflection data, where a composite kinematic pattern (i.e., transtension and transpression) would imply segmentation of the tectonic features.

If the excess of deformation is compensated by aseismic strain across creeping faults, could it be related to the crustal rheology?

This hypothesis is supported by some observations, such as the anomalous temperature structure, the presence of magmatic activity and the low crustal thickness (Fig. 6c). Surface heat flow measurements carried out in the last decades (http://www.datapages.com/gis-map-publishing-program/gis-open-files/global-framework/global-heat-flow-database) show values ranging from 50 to 100 mW m^−2^ in the Malta trough and in the Gela basin, while values up to 135 mW m^−2^ are observed in the Linosa and Pantelleria troughs and on the Adventure Bank (Fig. 6c). In addition, subaerial and submerged Plio-Pleistocene volcanoes^11–13^ are mainly located along the LSSZ. At crustal depth (15–25 km) these volcanic edifices correspond to some low P-wave velocity bodies^32^. Moreover, the estimated crustal thickness is ~ 21 km in the central part of the Sicily Channel, with increasing values up to 32 km both northward and southward^51^. All this evidence lends credit to a more ductile rheology across the LSSZ, which would significantly inhibit frictional sliding and favours creeping and aseismic deformation. Indeed, the LSSZ allows weak material ascent into the intraplate shear zone, and eventually migrating laterally to form strong lateral heterogeneities, both in composition and mechanical strength. Based on these observations, we favour the hypothesis that a considerable amount of the estimated crustal deformation-rate budget occurs aseismically, at least, in the northern sector of the Sicily Channel area. Moreover, under the higher confinement that exists deeper in the Earth’s lithosphere, brittle‐like (i.e. sudden, localized) failure may occur, as testified by observation of occasionally deep earthquakes along the LSSZ (Fig. 2a).

## Conclusions

A multidisciplinary analysis of geodetic, seismological and seismic reflection data provides an updated picture of active tectonic features in northern Sicily Channel region, a key area recording the Nubia–Eurasia plate interaction in the Mediterranean region. All analysed data, integrated by reconstructions and observations coming from literature, point to the presence of a N–S tectonic lineament we named the *Lampedusa—Sciacca shear zone* (LSSZ), which represents the most active tectonic domain in the study area and accounts for only ~ 0.9% of crustal deformation, as deduced by comparing geodetic and seismological moment-rate budgets. Our preferred scenario, supported by collateral evidences, such as incipient magmatism, high heat-flow and the reduced crustal thickness, points to a relatively ductile rheology of the crust, suggesting an aseismical restoration of this deficit. This implies a thorough re-evaluation of the seismic hazards in this region, where only a small portion of the inferred deformation would be compensated by minor to moderate future earthquakes.

## Data and methods

### Seismological data

We collected a catalogue of instrumental seismicity taking into account all data records reported in on-line bulletins (http://www.isc.ac.uk/iscbulletin/search/catalogue/; http://iside.rm.ingv.it). For the study area (Fig. 2a), we selected 1780 earthquakes covering the time interval 1966–2018, with magnitude between 1.5 and 5.5. Hypocentres collected from ISC bulletin span the 1966–1984 time interval. For the earthquakes of this period (~ 3% of the whole collected dataset), the bulletin does not provide uncertainties of location parameters, except for a few records, for which the mean error on horizontal coordinates is ~ 12 km. Records coming from the other bulletin (http://iside.rm.ingv.it) cover the period 1985–2018, and refer to earthquakes mainly acquired by the seismic network managed by Istituto Nazionale di Geofisica e Vulcanologia (INGV). Uncertainties of the hypocenter locations are, on average, 3, 6 and 2 km for longitude, latitude and depth coordinates, respectively. Nevertheless, numerous locations are reported with fixed focal depth, so they may suffer from greater uncertainties. Available historical seismic catalogues report, for the area in Fig. 2a, the occurrence of large earthquakes (M ≥ 6.5) since 1125 (https://www.emidius.eu/SHEEC/). The accuracy of these catalogues is not uniform and the epicentral location of some historical earthquakes may result uncertain, mainly due to the presence of wide sea areas and the sparsely populated region. This is the case of several earthquakes which are clustered closely to the main towns and villages, clearly reflecting the distribution of populated areas along the southern Sicilian coastal area and Pantelleria island where the shocks could be felt^27^.

The seismic moment-rate ($\dot{M}_{seis}$) has been calculated as^34^:

1 $$\dot{M}_{seis} = \phi \frac{b}{{\left( {c - b} \right)}}10^{{\left[ {\left( {c - b} \right)M_{x} + a + d} \right]}}$$

where *φ* is a correction for the magnitude (*M*)—moment (*M*_*seis*_) relation, *M*_*x*_ is the magnitude value of the largest earthquake that could occur within the investigated region, *c* and *d* (with values 1.5 and 9.1 for *M*_*seis*_ in Nm, respectively) are the coefficients of the relation^67^:

2 $$\log M_{seis} = cM + d$$

while *a* and *b* are the coefficients of the earthquake frequency–magnitude distribution^68^:

3 $$\log N\left( M \right) = a - bM$$

with *N(M)* the annual cumulative number of earthquakes having magnitude equals to or greater than *M*. The earthquake frequency–magnitude distribution breaks down at the value of *M*_*c*_ (magnitude of completeness), which theoretically defines the lowest magnitude at which 100% of the earthquakes in a space–time volume are detected^69^. To estimate the coefficients of the earthquake frequency–magnitude distribution, we defined a sub-catalogue by extracting from our instrumental catalogue only the earthquakes falling within the area outlined by the blue polygon in Fig. 2a. Such a sub-catalogue (468 events) covers the 1968–2018 interval with magnitude values between 1.5 and 4.6 (Fig. 2c). Finally, we calculated the *a, b* and *M*_*c*_ values for this sub-catalogue by using a maximum likelihood estimation technique^70^, obtaining values of 3.85 (± 0.11), 1.12 (± 0.08) and 2.8 (± 0.2), respectively (uncertainties at the 95% of confidence; Fig. 2d and Table 1).

Earthquake magnitudes in our data refer to different scales: *M*_*l*_ (local magnitude), *M*_*d*_ (duration magnitude), *m*_*b*_ (body wave magnitude) and *M*_*s*_ (surface wave magnitude). Ideally these magnitudes should be converted into moment magnitude (*M*_*w*_), which for the moment-rate calculation should be used as the standard scale, given the limitations of the other magnitude scales. Due to the lack of a regional relationship between the different scales, here we converted all earthquake magnitudes directly into scalar moments by using the above generalized relation. Finally, assuming *φ* = 1.71 (which reflects an average error of 0.3 on catalogue magnitudes^34^) and *M*_*x*_ = 5.7, we estimated a seismic moment-rate of 6.48 × 10^15^ Nm/year (see Table 1 for details on all parameters).

### Geodetic data

Raw GPS observations were reduced to loosely constrained daily solutions by using the GAMIT/GLOBK software packages^71^. The analysed dataset consists of data from up to 50 GPS stations (with more than 2.5 years of observations) belonging to various networks developed in the last two decades for crustal deformation studies and commercial applications (e.g., mapping and cadastral purposes). Estimated GPS velocities refer to a Nubian-fixed reference frame^37^.

In order to estimate the geodetic strain-rate, in a first step we derived a continuous velocity gradient over the study area on a regular 0.25° × 0.25° grid (with nodes not coinciding with any GPS stations) using a “spline in tension” function^72^ by using as input the horizontal velocity field and associated uncertainties. The tension is controlled by a factor T, where T = 0 leads to a minimum curvature (natural bicubic spline), while T = 1 allows for maxima and minima only at observation points^73^. We set T = 0.5 because such value represents the optimal to minimize the short wavelength noise^74^. Lastly, we computed the average strain-rate tensor as derivative of the velocities at the centres of each cell (Fig. 3). We also estimated the geodetic moment-rate $\dot{M}_{geod}$ which is defined as^75^:

4 $$\dot{M}_{geod} = 2\mu H_{s} A[Max(|\varepsilon_{H\max } |,|\varepsilon_{h\min } |,|\varepsilon_{H\max } + \varepsilon_{h\min } |)]$$

with *μ* the shear modulus of the rocks (taken here as 3·10^10^ N/m^2^; Table 1), *H*_*s*_ (seismogenic thickness) and *A* (surface area; 4.1·10^10^ m^2^ in our case study) define the seismogenic volume over which strain accumulates and its elastic part is released as earthquakes, *ε*_*Hmax*_ and *ε*_*hmin*_ are the principal geodetic horizontal strain-rates and *Max* is a function returning the largest of the arguments. In order to assess the robustness of our moment-rate estimations we performed some additional computations of the strain-rate field by simply varying, from 0.05° to 1.0°, the size of the computational grid (see Supplementary Information).

### Marine geophysical data

We analysed a set of SPARKER seismic reflection profiles acquired by the Istituto di Geologia Marina (now Istituto di Scienze Marine; ISMAR) in the Sicily Channel during the 70’s. These data, available only in hard copies, have been digitized, processed and geo-referenced using the open-source software Seisprho^76^. The seismic source was a 30 kJ Teledyne system, and the receiver was a single channel streamer with an active section of 50 m. Shot interval was 4–8 s, corresponding to a horizontal spacing of about 12–24 m. Among the available seismic profiles (Figs. 4 and 5), we selected those crossing the area characterized by higher instrumental seismicity.

## Supplementary information

Supplementary Information.
