# Supplementary material for: Crustal deformation, active tectonics and seismic potential in the Sicily Channel (Central Mediterranean), along the Nubia–Eurasia plate boundary
Source: Sci Rep. 2020 Dec 4;10:21238. doi: 10.1038/s41598-020-78063-1 (PMC7719175; doi:10.1038/s41598-020-78063-1)
Supplement: Supplementary file 1 — Supplementary Information. [file 41598_2020_78063_MOESM1_ESM.docx]

**Crustal deformation, active tectonics and seismic potential in the Sicily Channel (Central Mediterranean), along the Nubia-Eurasia plate boundary**

**Supplementary Information**

Mimmo Palano^1^, Andrea Ursino^1^, Salvatore Spampinato^1^, Federica Sparacino^1^, Alina Polonia^2^, Luca Gasperini^2^

1 - Istituto Nazionale di Geofisica e Vulcanologia, Sezione di Catania - Osservatorio Etneo, Piazza Roma 2, I-95125, Catania, Italy

2 - Institute of Marine Sciences CNR ISMAR-Bo, Via P. Gobetti 101, I-40129, Bologna, Italy

**1 Background setting of the Sicily Channel**

The Sicily Channel is part of the Pelagian block^1,2,3^, extending from the Sahel region of Tunisia to eastern Sicily. It is separated from the Ionian basin by a regional tectonic boundary named the Hyblean-Maltese Escarpment fault system (Fig. 1). The tectonic configuration of the Pelagian block consists of a series of mostly WNW-ESE trending structural highs and basins, bordered by variously oriented faults of Neogene-Quaternary age. The autochthonous sedimentary wedge (ca. 7-8 km thick) consists of Triassic to Pleistocene carbonate deposits, with intercalated volcanoclastic units and minor lava flows covered by Upper Tortonian-Lower Messinian siliciclastic deposits and Plio-Quaternary clastic sequences^4^.

Evolution of the Pelagian block has been strongly influenced by the complex Nubia-Eurasia plate interaction. From the Late Cretaceous to Miocene, a convergence pattern is reported along directions varying from NE-SW to N-S between the eastern and the western Mediterranean Sea^5^. The plate convergence direction shifted to NW-SE around ∼7Ma^6^ (Early Messinian) remaining stable since then. Such an orientation change would correspond with i) the decoupling of the Adriatic promontory from the Nubian plate through the activation of a major discontinuity in the Ionian basin, and ii) the Adria plate clockwise rotation with respect to Eurasia, leading to the consumption of a large part of the Ionian Tethys and to a roughly northward expulsion of the Pelagian block^7^. Since then, the Pelagian block has acted as the foreland of the Sicilian-Maghrebian thrust belt, a collisional orogeny originated from the Nubia and Eurasia convergence^8,9^. In addition, starting from the Late Miocene, and mostly during the Early Pliocene, the central part of the Pelagian block was affected by a lithospheric-scale continental rifting process, followed in the Late Pliocene-Pleistocene by a phase characterized by a magma-assisted extensional mechanism^10^. Cutting the Miocene thrust system^11^, the rifting process led to the development of NW-SE-oriented tectonic depressions, such as the Pantelleria, Linosa and Malta troughs^12^ (Fig. 1).

Tectonic bathymetric, volcanic and magnetic lineaments allow defining a N-S oriented belt, extending from Lampedusa to the Graham Bank^13,14,15^ (Fig. 1). This belt separates the rift system in two sectors: the Pantelleria trough to the west, and the Malta and Linosa troughs to the east (Fig. 1).

The volcanoes of the Sicily Channel (Linosa and Pantelleria islands and numerous seamounts; Fig. 1) are mainly located along the NW-SE trending faults bordering the rift system and along the N-S oriented belt. Previous studies^16,17^ suggested that magmatic activity began in the Nameless Bank during the Late Miocene and continued to the present-day near Pantelleria Island and the southern wedge of the Graham Bank (Fig. 1). An intense volcanic activity has been observed in the region during historical times, including a submarine volcano on the Graham Bank that emerged during an eruption in 1831 giving rise to an ephemeral island, the Ferdinandea Island^18^, as well as two eruptions in the Sicily offshore in 1845 and 1846. A submarine volcanic eruption occurred in 1891 ~5 km northwest of Pantelleria, while vigorous gas emissions were observed in 1941 at the southeastern wedge of Graham Bank^19,20^. Pantelleria island volcanic rocks consist of peralkaline trachytes and rhyolites (pantellerites) and minor Na-transitional to mildly alkaline basalts^21^. Linosa rocks range in composition from mildly alkaline basalts to hawaiite, while rock compositions of seamounts range from tholeiitic basalt to alkali basalt, hawaiite and basanite^21^.

**2 Strain-rate tests**

In order to assess the robustness of our moment-rate estimations we performed some additional computations of the strain-rate field by simply varying the size of the computational grid (from 0.05° to 1.0°; see Fig. S1).


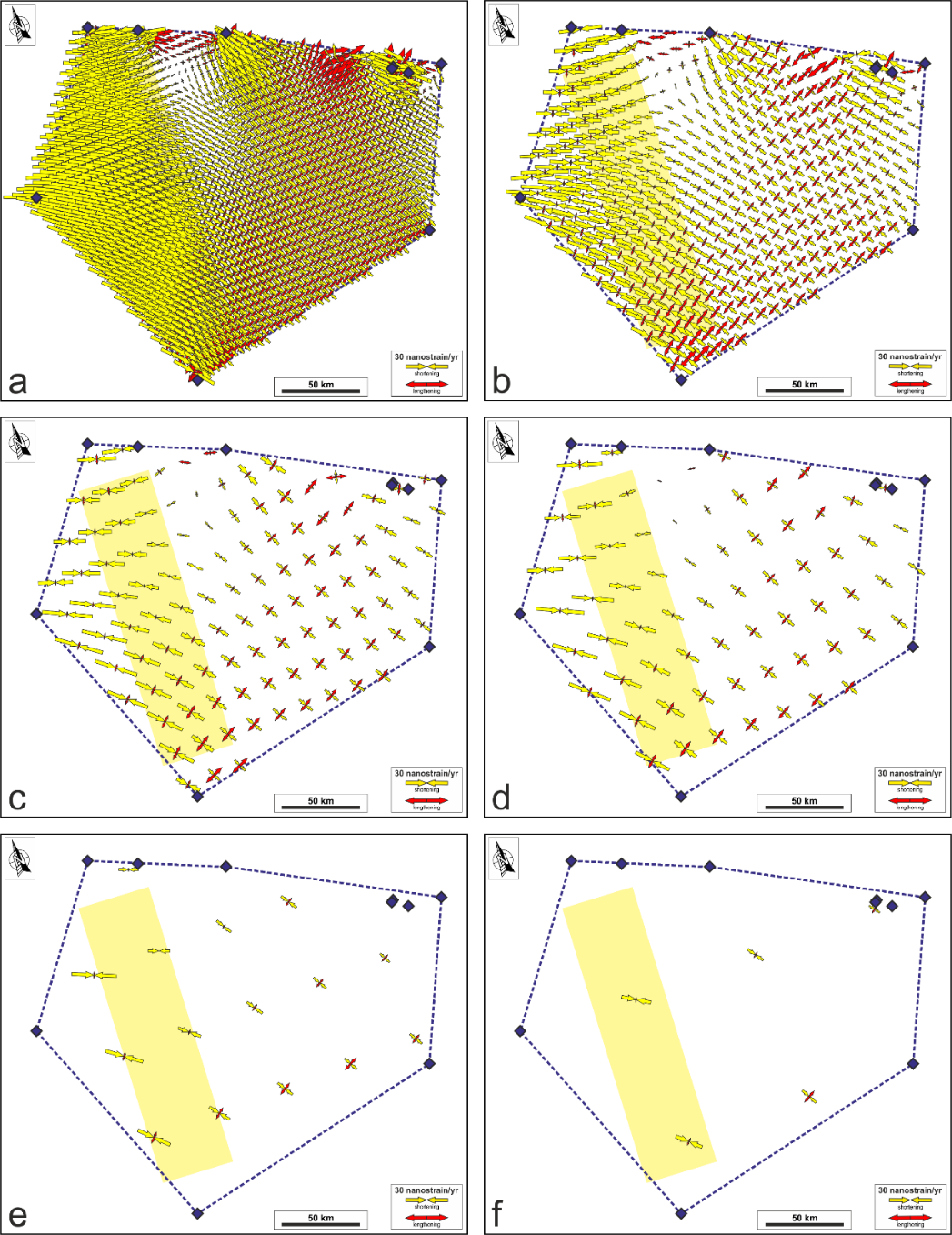


**Figure S1.** Examples of strain-rate fields computed by using different grid size: a) 0.05° x 0.05°, b) 0.10° x 0.10°, c) 0.20° x 0.20°, d) 0.25° x 0.25°, e) 0.50° x 0.50°, f) 1.0° x 1.0°. The yellow strip represents the N-S-oriented tectonic belt. The blue polygon defines the study area. The blue diamonds represent the continuous GPS stations. Maps compiled using the Generic Mapping Tool, version 5 (https://www.generic-mapping-tools.org); image editing using Inkscape, version 1 (https://inkscape.org).


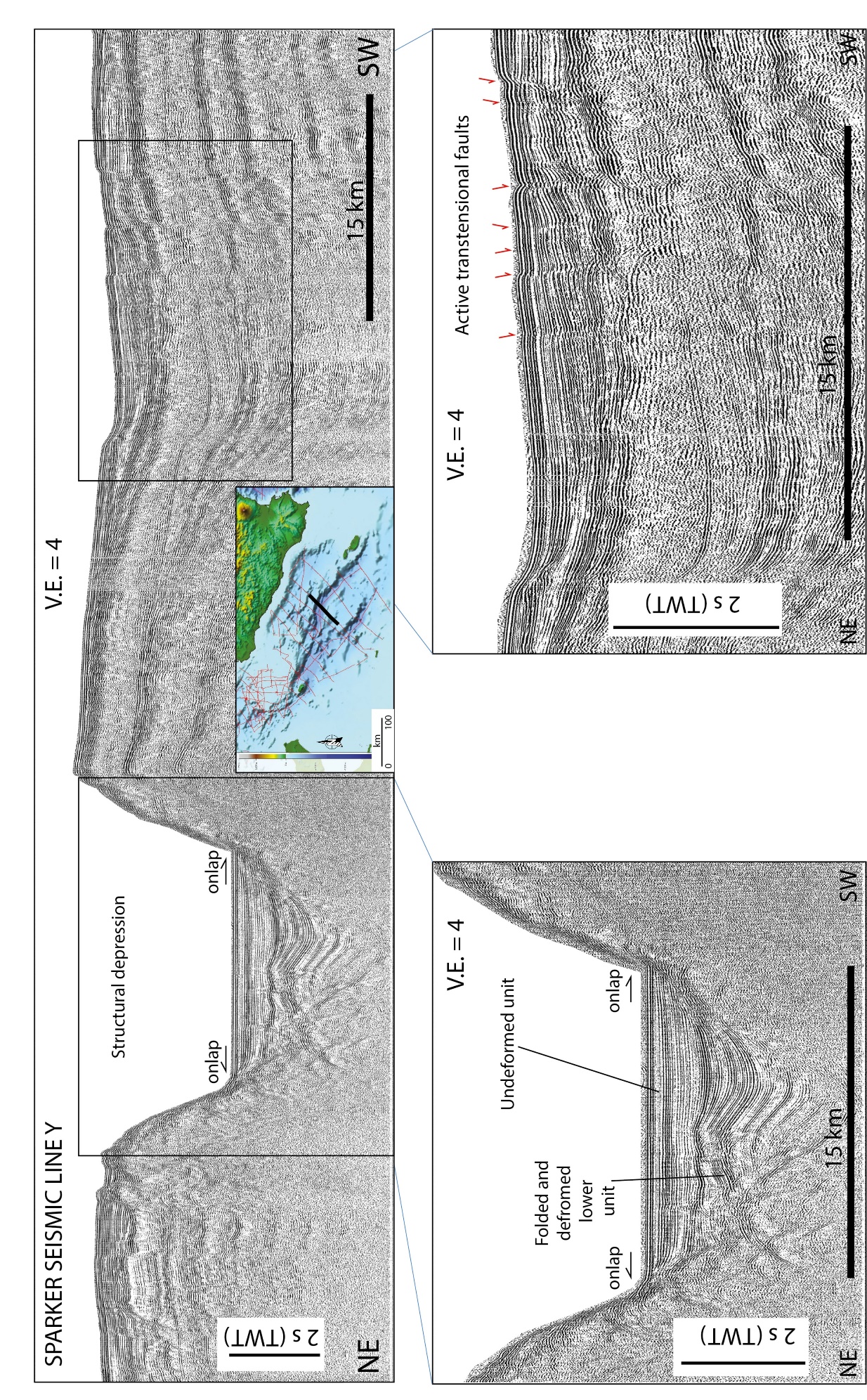


**Figure S2.** Seismic profile Y crossing orthogonally the deep trough of the Sicily Channel. See Fig. 4 for geological interpretation. The images have been edited by using Adobe Illustrator CS6.


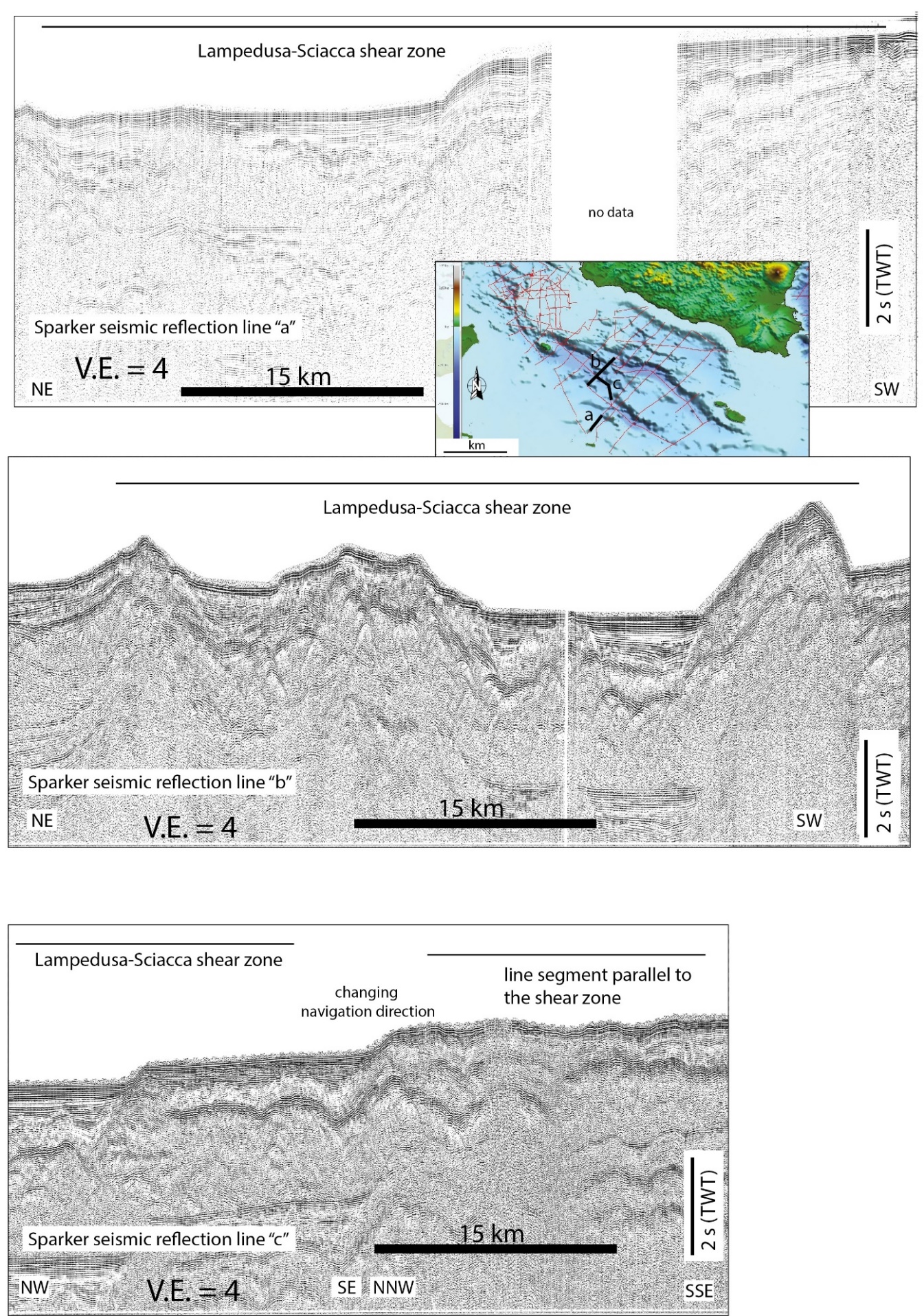


**Figure S3.** Seismic reflection profiles along the N-S corridor where strike-slip deformation has been detected. See Fig. 5 for geological interpretation. The images have been edited by using Adobe Illustrator CS6.


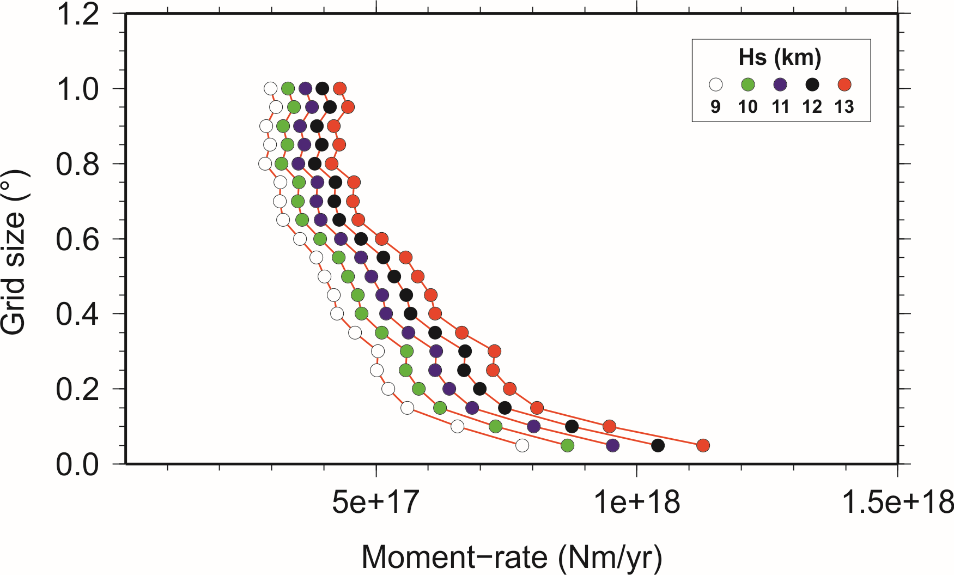


**Figure S4.** a) Variation of geodetic moment-rate with respect to the size of the computational grid and the seismogenic thickness *H_s_*. Maps compiled using the Generic Mapping Tool, version 5 (https://www.generic-mapping-tools.org).

**References**

1. Burollet, P. F., Mugniot, J. M. & Sweeney, P. The geology of the Pelagian block: the margins and basins of southern Tunisia and Tripolitania in *The ocean basins and margins. The Western Mediterranean* (eds. Nairn, A. E. M., Kanes, W. H. & Stelhi, F. G.) 331-359 (Plenum, New York, 1978).
2. Ben-Avraham, Z. & Grasso, M. Crustal structure variations and transcurrent faulting at the eastern and western margins of the eastern Mediterranean. *Tectonophysics* **196**(3-4), 269-277 (1991).
3. Lentini, F., Carbone, S., Guarnieri, P., Dilek, Y. & Pavlides, S. Collisional and postcollisional tectonics of the Apenninic-Maghrebian orogen (southern Italy). *Spec. Pap. Geol. Soc. Am.* **409**, 57 (2006).
4. Torelli, L., Grasso, M., Mazzoldi, G., Peis, D. & Gori, D. Cretaceous to Neogene structural evolution of the Lampedusa shelf (Pelagian Sea, Central Mediterranean). *Terra Nova* **7**(2), 200-212 (1995).
5. Dewey, J. F., Helamn, M. L., Turco, E., Hutton, D. H. W. & Knott, S. D. Kinematics of the western Mediterranean in *Alpine Tectonics, Geological Society* (eds. Coward, M. P., Dietrich, D. & Park, R. G.) 265-283 (London, Special Publication 45, 1989).
6. DeMets, C., Iaffaldano, G. & Merkouriev, S. High-resolution Neogene and Quaternary estimates of Nubia-Eurasia-North America Plate motion. *Geophys. J. Int.* **203**, 416-427 (2015).
7. Mantovani, E. *et al*. Generation of back-arc basins as side effect of shortening processes: Examples from the Central Mediterranean. *Int. J. Geosci.* **5**(10), 1062 (2014).
8. Ben Avraham Z., Boccaletti M., Cello G., Grasso M., Lentini F., Torelli L. & Tortorici L. Principali domini strutturali originatisi dalla collisione neogenico-quaternaria nel Mediterraneo Centrale. *Mem. Soc. Geol. Ital.* **45**, 453-462 (1990).
9. Lentini, F., Carbone, S., Guarnieri, P., Dilek, Y. & Pavlides, S. Collisional and postcollisional tectonics of the Apenninic-Maghrebian orogen (southern Italy). *Spec. Pap. Geol. Soc. Am.* **409**, 57 (2006).
10. Civile, D. *et al*. The Pantelleria graben (Sicily Channel, Central Mediterranean): an example of intraplate ‘passive’rift. *Tectonophysics* **490**(3-4), 173-183 (2010).
11. Casero, P. & Roure, F. Neogene deformations at the Sicilian-North Africa plate boundary in *Peri-Tethyan Platforms* (ed. Roure, F.) 27-45 (Technip, Paris, 1994).
12. Boccaletti, M., Cello, G. & Tortorici, L. Transtensional tectonics in the Sicily Channel. *J. Struct. Geol.* **9**(7), 869-876 (1987).
13. Cello, G. Structure and deformation processes in the Strait of Sicily “rift zone”. *Tectonophysics* **141**, 237-247 (1987).
14. Argnani, A. The strait of Sicily rift zone: Foreland deformation related to the evolution of a back-arc basin. *J. Geodyn.* **12**, 311-331 (1990).
15. Lodolo, E., Civile, D., Zanolla, C. & Geletti, R. Magnetic signature of the Sicily Channel volcanism. *Mar. Geophys. Res.* **33**(1), 33-44 (2012).
16. Beccaluva, L., Colantoni, P., Di Girolamo, P. & Savelli, C. Upper Miocene submarine volcanism in the Strait of Sicily (Banco Senza Nome). *Bull. Volcanol.* **44**, 573-581 (1981).
17. Cavallaro, D. & Coltelli, M. The Graham volcanic field offshore southwestern Sicily (Italy) revealed by high-resolution seafloor mapping and ROV images. *Front. Earth Sci.* (2019).
18. Corti, G., Cuffaro, M., Doglioni, C., Innocenti, F. & Manetti, P. Coexisting geodynamic processes in the Sicily Channel. *Special Papers-Geol. Soc. Am.* **409**, 83 (2006).
19. Carapezza, M., Ferla, P., Nuccio, P. M. & Valenza, M. Caratteri petrologici e geochimici delle vulcaniti dell’Isola Ferdinandea. *Rend. Soc. Ital. Mineral. Petrol.* **35**(1), 377-388 (1979).
20. Spampinato, S. *et al*. A reappraisal of seismicity and eruptions of Pantelleria Island and the Sicily Channel (Italy). *Pure Appl. Geophys.* **174**, 2475-2493 (2017).
21. Peccerillo, A. The Sicily Province in *Plio-quaternary volcanism in Italy* (ed. Peccerillo, A.) 215-255 (Springer-Verlag, Berlin 2005).
